# Society Meetings

**Published:** 1913-10

**Authors:** 


### SOCIETY MEETINGS.

*Brief reports and announcements of meetings of societies of Western New York and of those of general scope are requestedm from Secretaries. Copy should be on hand by the fifteenth of each month. Full transactions will be published at cost of composition.*

The third bi-ennial meeting of the POLISH PHYSICIANS ASSOCIATION OF AMERICA was held on September 19th at Hotel Cadillac, Detroit, Mich. Dr. Francis E. Fronczak, Health Commissioner, Buffalo, N. Y., President, presiding.

Over fifty members from all over the country were present. The association numbers about 250.

At the business meeting, at which various reports were received, action was taken whereby the Polish press of this country will be asked to refuse to accept advertising matter from quacks and physicians who, under the guise of free medical advisors, etc., have been demoralizing the Polish people and at the same time reaping a financial harvest. The Polish press of this country has been flooded with this kind of advertising, and the Polish press can aid in suppressing this great fraud.

A complete directory of the Polish physicians of the United States is soon to be compiled. Matters referring to the protection of the profession, also the matter of helping the struggling medical student, the establishment of headquarters for the Polish

Physicians' Associations of America, where all information can be gathered and distributed to the profession and to the student were acted on.

Papers were read and discussed by Dr. Pietrzykowski of Chicago; Dr. R. Sadowski of Detroit; Dr. Zurawski of Chicago, Dr. Wagner of Milwaukee; Dr. Krajewski of Nanticoke, Dr. Francis E. Fronczak of Buffalo, Dr. Ostrowski of Hammond, Ind.; Dr. Peters of Cleveland, Dr. Ratajski of Shenandoah, and many others.

The following officers were elected: Dr. Francis E. Fronczak, President, Buffalo; Dr. Kas. Zurawski, Vice-President, Chicago; Dr. S. N. Borowiak, Secretary, Buffalo; Dr. Eug. Koneczny, Treasurer, Detroit.

---

The Medical Society of the County of Chemung held its regular fall meeting at Elmira, September 16. Dr. C. S. Carey read a paper on "The Correction of Eye Strain in Functional Nervous Diseases," and Dr. H. W. Fudge gave a case report.

---

The Buffalo Medical and Surgical League met at the Hotel Statler, September 11, a symposium being held.

---

The Seventh and Eighth District Branches of the State Society held a joint meeting at Sonyea, September 24 and 25. A number of guests were entertained at the State Institution over night, in order that the business meeting of the Eighth Branch might be held on the evening of September 24. The program was as follows:

President's Address, "The Eighth District Branch," Arthur G. Bennett, M. D., Buffalo; President's Address, "The Seventh District Branch," William T. Shanahan, M. D., Sonyea; "The Burden of Mental Defect," Herman G. Matzinger, M. D., Buffalo; "Field Work in the Study of Epilepsy," David F. Weeks, M. D., Supt. New Jersey State Village for Epileptics; Discussion opened by Dr. Gertrude E. Hall, Bureau of Analysis, New York State Board of Charities; "Recent Advances in Neurology and Psychiatry," Edward L. Hanes, M. D., Rochester; Discussion opened by Eveline P. Ballintine, M. D., Rochester; "Landry's Paralysis and Its Relation to Acute Poliomyelitis." Edward A. Sharp, M. D., Buffalo; Discussion opened by Nelson G. Russell, M. D., Buffalo; Recess for luncheon; "The History of Surgical Intervention in Epilepsy," Roswell Park, M. D., Buffalo; "The Association of Skin Lesions with Diabetes," John R. Williams, M. D., Rochester; "Suggestions for a New Classification of the Syphilides" (illustrated by stereopticon), Grover W. Wende, M. D., Buffalo; "Sugar Tolerance in Epilepsy" (illustrated by stereopticon), James F. Munson, M. D., Sonyea; Discussion opened by Arthur L. Shaw, M. D., Sonyea; "A Further Report Upon Some Hematological Cases," John M. Swan, M. D., Rochester; "Intravenous Use of Paraldehyde," G. Kirby Collier, M. D., Sonyea.

---

THE CAYUGA COUNTY MEDICAL SOCIETY held its quarterly meeting on September 11. Dr. Wm. F. Campbell, President of the W. Society of the State of New York, was present and addressed the meeting.

